# Identification of novel monocistronic HTLV-1 mRNAs encoding functional Rex isoforms

**DOI:** 10.1186/s12977-015-0184-2

**Published:** 2015-07-02

**Authors:** Francesca Rende, Ilaria Cavallari, Vibeke Andresen, Valerio W Valeri, Donna M D’Agostino, Genoveffa Franchini, Vincenzo Ciminale

**Affiliations:** Department of Surgery, Oncology and Gastroenterology, University of Padova, Padua, Italy; Animal Models and Retroviral Vaccines Section, National Cancer Institute, Bethesda, MD USA; Department of Biomedical Sciences, University of Padova, Padua, Italy; Istituto Oncologico Veneto-IRCCS, Padua, Italy; Translational Hemato-Oncology Group, Department of Clinical Science, Centre of Cancer Biomarkers CCBIO, University of Bergen, Bergen, Norway; Novartis Vaccines Loc., Bellaria Rosia, 53018 Sovicille, SI Italy

**Keywords:** HTLV-1, Rex, Splicing

## Abstract

**Background:**

Human T cell leukemia virus type 1 (HTLV-1) gene expression is controlled by the key regulatory proteins Tax and Rex. The concerted action of these proteins results in a two-phase kinetics of viral expression that depends on a time delay between their action. However, it is difficult to explain this delay, as Tax and Rex are produced from the same mRNA. In the present study we investigated whether HTLV-1 may produce novel mRNA species capable of expressing Rex and Tax independently.

**Findings:**

Results revealed the expression of three alternatively spliced transcripts coding for novel Rex isoforms in infected cell lines and in primary samples from infected patients. One mRNA coded for a Tax isoform and a Rex isoform, and two mRNAs coded for Rex isoforms but not Tax. Functional assays showed that these Rex isoforms exhibit activity comparable to canonic Rex. An analysis of the temporal expression of these transcripts upon ex vivo culture of cells from infected patients and cell lines transfected with a molecular clone of HTLV-1 revealed early expression of the dicistronic *tax/rex* mRNAs followed by the monocistronic mRNAs coding for Rex isoforms.

**Conclusion:**

The production of monocistronic HTLV-1 mRNAs encoding Rex isoforms with comparable activity to canonical Rex, but with distinct timing, would support a prolonged duration of Rex function with gradual loss of Tax, and is consistent with the two-phase expression kinetics. A thorough understanding of these regulatory circuits will shed light on the basis of viral latency and provide groundwork to develop strategies for eradicating persistent infections.

## Findings

**Figure 1:**
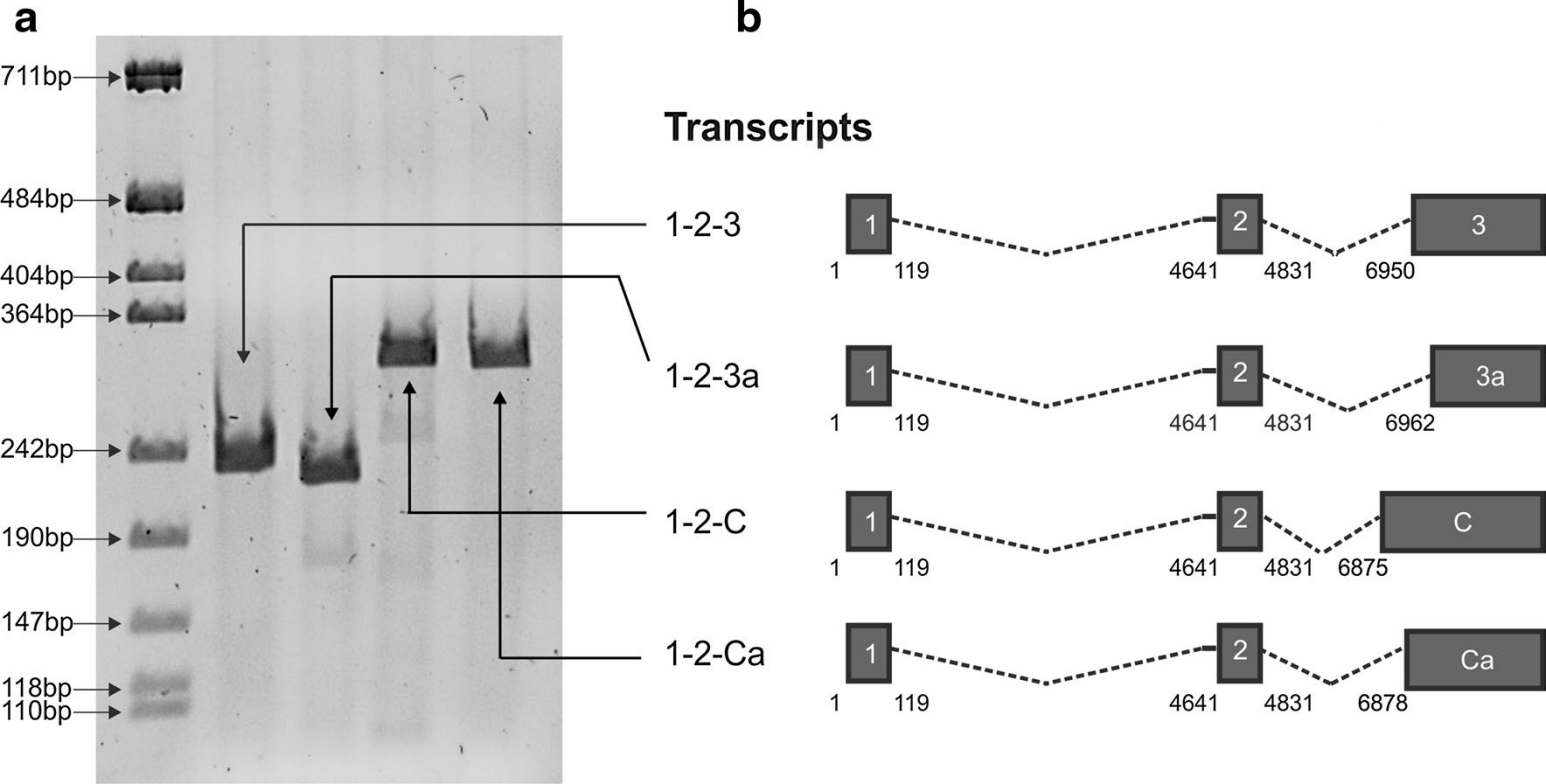
Identification of novel alternatively spliced HTLV-1 mRNAs. **a** RT-PCR analysis of doubly spliced mRNAs produced in HLtat cells transfected with a plasmid containing the ACH molecular clone [9]. RT-PCR was carried out with primers (Table 1) to detect transcripts joining exon 2 to the splice acceptor (SA) site of exon 3 (nucleotide 6950) or alternative to exon 3 (nucleotides 6875, 6878 and 6962). **b** Exon composition of the canonical (*1*-*2*-*3*) mRNA and of the three novel mRNAs (*1*-*2*-*3a*, *1*-*2*-*C* and *1*-*2*-*Ca*). The Hela-derived cell line HLtat [10] was maintained in Dulbecco’s modified Eagle’s medium (Sigma-Aldrich) supplemented with 10% fetal calf serum (Invitrogen), 100 units/mL penicillin, and 20 units/mL streptomycin. HLtat cells were seeded into 35 mm cell culture plates at 1.5 × 10^5^ cells/plate and transfected 1 day later with 1 µg of the ACH plasmid using GeneJuice Transfection Reagent (Novagene, Merck Millipore) and harvested 24 h after transfection for RNA extraction, DNAase treatment, reverse-transcription and PCR analysis (RT-PCR) as previously described [5].

Human T cell leukemia virus type 1 (HTLV-1) is the causative agent of adult T-cell leukemia-lymphoma (ATLL) and tropical spastic paraparesis/HTLV-1-associated myelopathy (TSP/HAM) (reviewed in [1]). HTLV-1 expression is controlled by Tax and Rex, two key viral regulatory proteins coded by a doubly-spliced dicistronic mRNA containing exons 1, 2 and 3 (Figure 1). Tax provides a positive feedback circuit by driving transcription of the viral genome [2]; Rex enhances the nuclear export and expression of a subset of mRNAs coding for the virion-associated proteins Gag-Pol and Env, while reducing the levels of the *tax/rex* mRNA, thus exerting a negative feedback loop on viral transcription [3, 4]. Using quantitative RT-PCR (qRT-PCR), we recently demonstrated a two-phase kinetics of HTLV-1 expression in short-term cultures of primary PBMCs from infected patients [5–7]. We also showed that this timing of viral gene expression was strictly dependent on Rex. Mathematical modelling [5, 8] indicated that a time delay between the positive (Tax) and negative (Rex) regulatory loops is necessary to explain the observed kinetics. This notion is in apparent contrast with the fact that Tax and Rex are known to be produced together from the *1*-*2*-*3* mRNA. However, we provided experimental evidence that Tax protein is more rapidly degraded than Rex, a property that might contribute, at least partially, to a temporal separation between maximal Tax and Rex function [5]. To better define the molecular mechanisms determining the time delay between Tax and Rex function, in the present study we investigated whether HTLV-1 may produce novel mRNA species capable of expressing Rex and Tax independently.

### Identification and coding potential of novel alternatively spliced HTLV-1 mRNAs

To determine whether HTLV-1 may produce novel Rex- and/or Tax-encoding mRNAs, we carried out a pilot in silico analysis to search for candidate splice acceptor (SA) sites in the vicinity of the canonical exon 3 SA at nt 6950. We focused our attention on potential sites defining 5′ exon boundaries located at positions 6875, 6878 and 6962, and performed RT-PCR with primers (Table 1) to detect transcripts joining exon 2 (which contains the Rex and Tax initiation codons) to these potential SA. Results showed that mRNAs spliced at these sites were produced in cells transfected with the HTLV-1 molecular clone ACH (Figure 1). Position 6875 was previously described as the exon C SA in the context of a singly spliced mRNA coding for p13 [11, 12], while the other SA sites were not previously described. As these sites are located in the vicinity of the SA for exon C and exon 3, we propose to name these sites Ca (6878) and 3a (6962), respectively.

**Table 1:** RT-PCR and qPCR primers and probes

RT-PCR and real time PCR primers
TaxRex s: 5′-GTCCGCCGTCTAG^CTTCC-3′ (exon 1^2 SA)
TaxRex as: 5′-CTGGGAAGTGGG^CCATGG-3′ (exon 2^3 SA)
Tax_a_Rex_a_ s: 5′-ACCACCAACACCATGG^GGTTTG-3′ (exon 2^3a SA)
Rex_b_ s: 5′-ACCACCAACACCATGG^CAGGTC-3′ (exon 2^C SA)
Rex_c_ s: 5′-ACCACCAACACCATGG^GTCCTC-3′ (exon 2^Ca SA)
Rex isoforms as: 5′-GAGTCGAGGGATAAGGAAC-3′
Real time probes
TaxRex: 5′ (FAM)-CCCAGTGGATCCCGTGGAG-3′(TAMRA)
Rex isoforms: 5′(FAM)-AAGGCGACTGGTGCCCCATCTCTGGG-3′(TAMRA)

**Figure 2:**
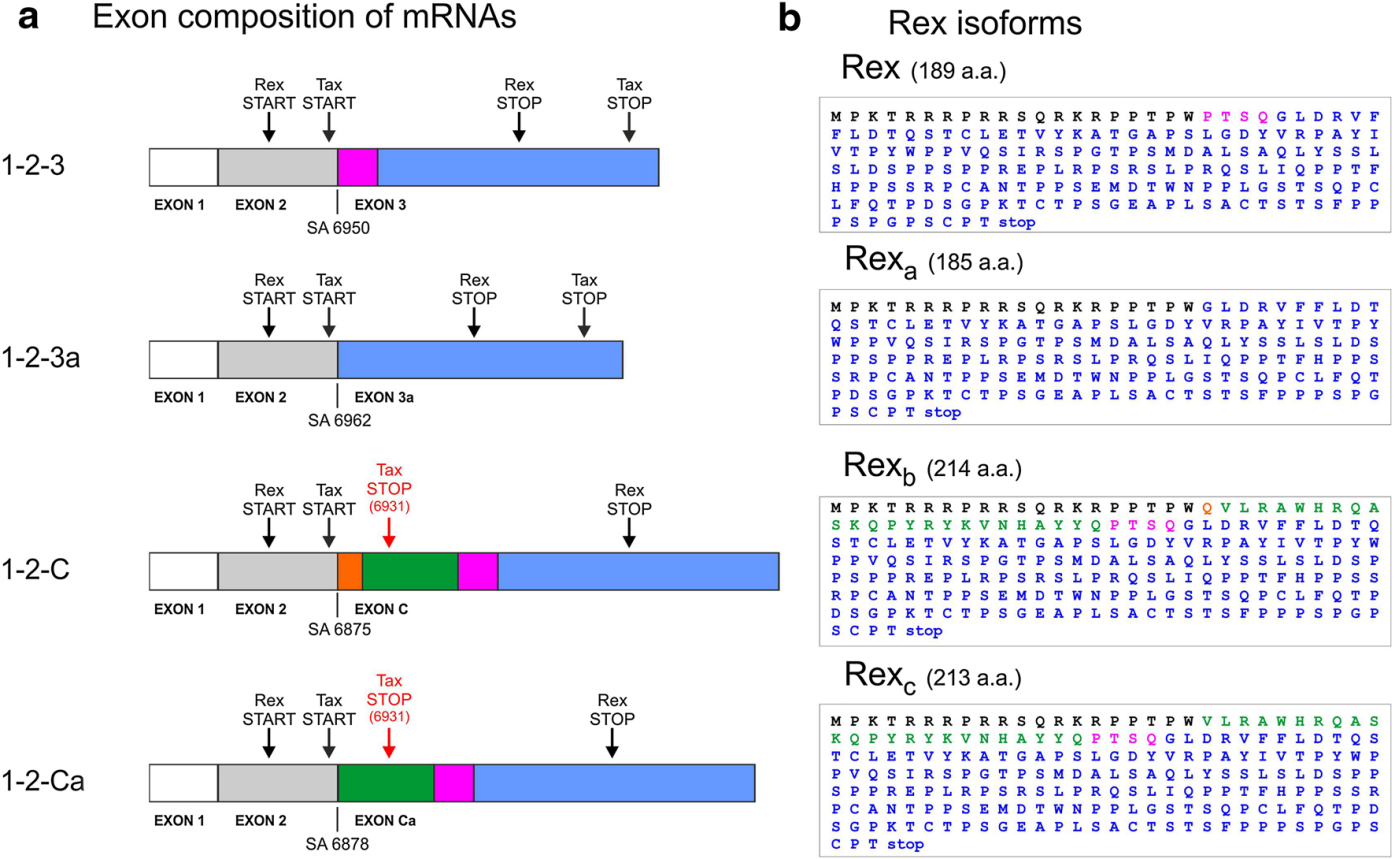
Coding potential of the novel mRNA species encoding Rex-protein isoforms. **a** Schematic representation of *1*-*2*-*3*, *1*-*2*-*3a*, *1*-*2*-*C* and *1*-*2*-*Ca* mRNAs; start and stop codons are indicated by *arrows*. *Coloured boxes* (not in scale) indicate the amino acid sequences differing in the alternatively spliced mRNAs. **b** Predicted amino acid sequences of proteins coded by the x-III ORF in the different mRNAs. Amino acid sequences are indicated in *colours* matching the *boxes* indicated in **a.**

Figure 2 shows the coding potential of these new transcripts. mRNA *1*-*2*-*3a* has the potential to encode 4-amino acid-shorter isoforms of both Tax and Rex, which we propose to term Tax_a_ and Rex_a_. The mRNAs *1*-*2*-*C* and *1*-*2*-*Ca* encode longer isoforms of Rex, which we propose to name Rex_b_ and Rex_c_, respectively, but are not predicted to produce functional Tax, as the x-IV ORF is truncated by a premature stop codon located at position 6931 (Figure 2). The sequence differences of Rex_a_, Rex_b_ and Rex_c_ (compared to Rex) are adjacent to the nuclear localization signal and upstream the multimerization domain of Rex [3, 13].

### Quantitation of novel alternatively spliced HTLV-1 mRNAs

**Figure 3:**
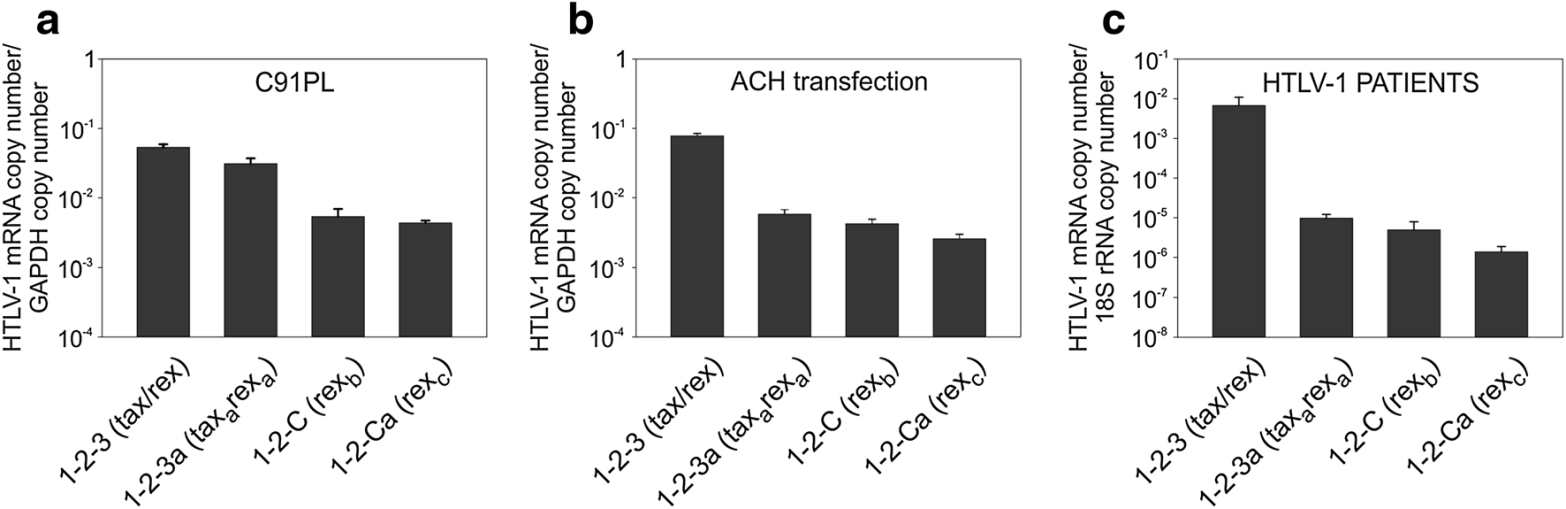
Quantitation of novel alternatively spliced HTLV-1 mRNAs. The *tax/rex* (*1*-*2*-*3*), *tax*_*a*_ */rex*_*a*_ (*1*-*2*-*3a*), *rex*_*b*_ (*1*-*2*-*C*) *and rex*_*c*_ (*1*-*2*-*Ca*) mRNAs were quantified by qRT-PCR in **a** the C91PL T-cell line, which is chronically infected with HTLV-1, **b** HLtat cells 24 h after transfection with the ACH molecular clone, and **c** Peripheral blood mononuclear cells (PBMC) from three HTLV-1-infected patients (ATLL-2, TSP-3 and TSP-4), after 48 h of culture in vitro. **a**, **b** Mean values and standard error bars from three independent experiments. C91PL cells [14] were maintained in RPMI 1640 (Sigma-Aldrich) supplemented with 10% FCS, 2 mM l-glutamine (GIBCO) and penicillin/streptomycin. PBMCs were isolated from peripheral blood samples donated by patients with a clinical diagnosis of ATLL or TSP/HAM attending the clinic at the National Centre for Human Retrovirology, Imperial College Healthcare NHS Trust, St. Mary’s Hospital or King’s College Hospital, London, UK, and treated as previously described [5, 15]. RNA extraction, DNAase treatment, reverse-transcription and qRT-PCR for the detection of HTLV-1 transcripts were performed as previously described [5] by using the primers and probes listed in Table 1. The absolute copy number of each transcript was determined and normalized (normalized copy number, NCN) for the copy number of 18S rRNA (patients’ samples) or GAPDH (C91PL and ACH transfections).

We next employed qRT-PCR to measure the levels of expression of these transcripts in the HTLV-1-infected cell line C91PL (Figure 3a), in the HeLa-derived cell line HLtat transfected with the HTLV-1 molecular clone ACH (Figure 3b), and in PBMCs from HTLV-1-infected ATLL and TSP-HAM patients (Figure 3c). The novel transcripts were detected in all of these samples. In C91PL cells the *1*-*2*-*3a* transcript was detected at levels similar to that of *1*-*2*-*3*, while the *1*-*2*-*C* and *1*-*2*-*Ca* transcripts were less abundant. In ACH-transfected HLtat and PBMC samples, all three novel transcripts were less abundant than the canonical *1*-*2*-*3 tax/rex* mRNA.

### Intracellular localization of Rex protein isoforms

**Figure 4:**
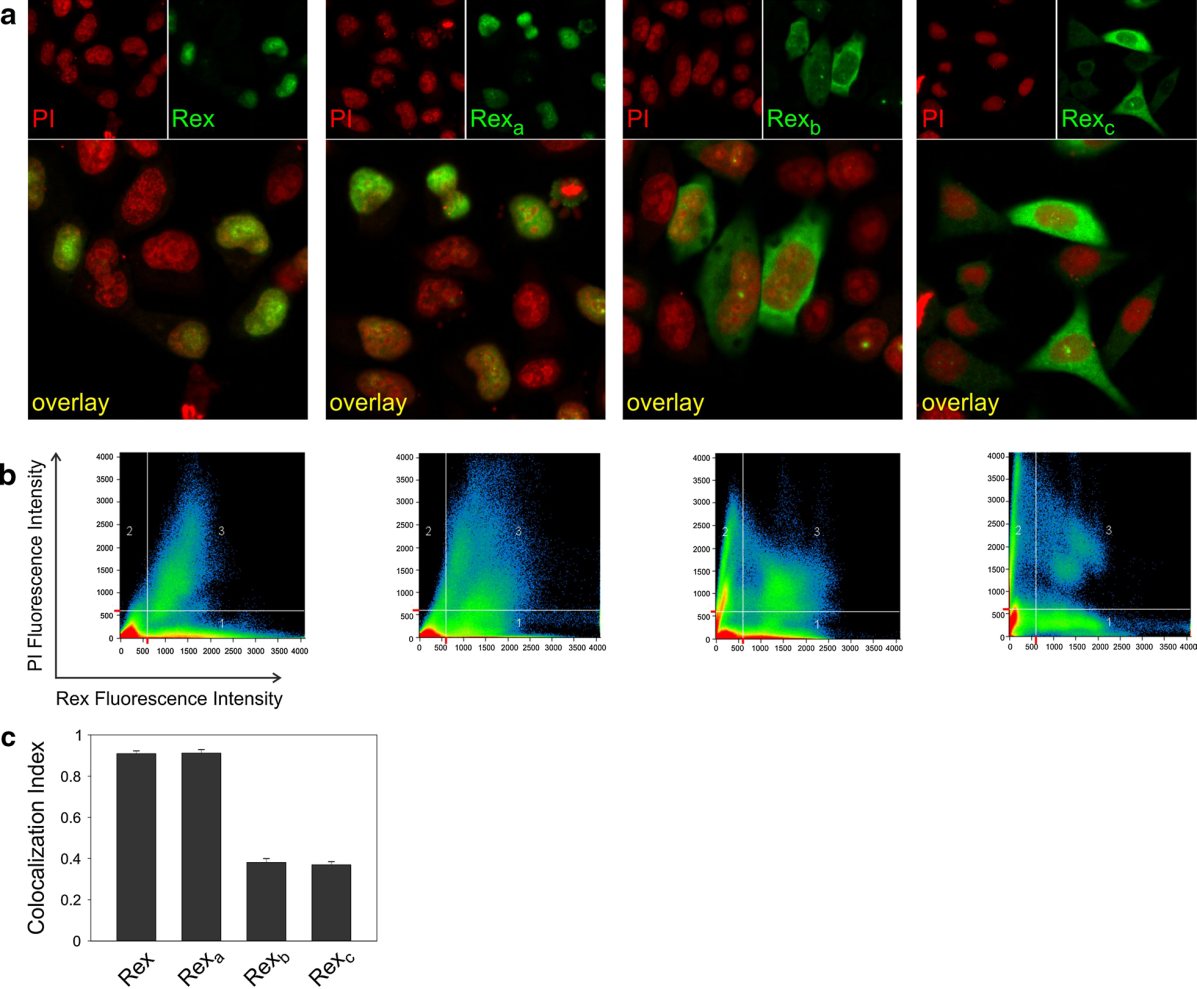
Intracellular localization of Rex protein isoforms. **a** IF/LSM of Rex isoforms in HLtat cells transfected with pMH-Rex, pMH-Rex_a_, pMH-Rex_b_ and pMH-Rex_c_ plasmids. The Rex signal is visualized in *green*, and propidium iodide (PI), used as a nuclear marker, is visualized in *red*. **b** Quantitative analysis of the Rex (X axis) and PI (Y axis) fluorescence per pixel (represented by *dots* in the scatter plot). **c** The ‘‘Histogram’’ software tool was used to measure the Colocalization Index (CI), indicating the fraction of Rex-positive pixels that were also PI-positive; *bars* represent mean CI values and standard error bars for at least 50 cells. Plasmids encoding Rex, Rex_a_, Rex_b_ or Rex_c_ (termed pMH-Rex, pMH-Rex_a_, pMH-Rex_b_ and pMH-Rex_c_, respectively) were generated by recombinant PCR to join exon 2 to exons 3, 3a, C and Ca, respectively. The resulting products were cloned in the expression vector pMHneo (Stratagene). HLtat cells were transfected with 1 µg of pMH-Rex, pMH-Rex_a_, pMH-Rex_b_ or pMH-Rex_c_. At 40 h after transfection, the cells were fixed in 4% formaldehyde (added to the culture medium) for 20 min, permeabilized with phosphate-buffered 0.1% NP40 for 10 min and treated with 100 µg/ml RNase for 1 h at room temperature. Cells were then incubated with a rabbit antibody (1:500) raised against amino acids 98–111 of Rex [16] for 1.5 h at 37°C, followed by incubation with an Alexa 488-conjugated anti-rabbit antibody (1:1,000, Molecular Probes) for 1 h at 37°C. Cells were then stained with 500 nM PI for 15 min at room temperature. Images were acquired with a Zeiss LSM 510 microscope using the Argon and Helium–Neon lasers (×63 optical magnification, ×4 scanning magnification). All parameters were standardized to allow comparison of signals obtained in different samples. Fluorescence signals were analyzed using a 505- to 530-nm band-pass filter for Alexa 488 and a long-pass 560-nm filter for PI.

Plasmids encoding Rex, Rex_a_, Rex_b_ and Rex_c_ were constructed and transfected into HLtat cells. Immunofluorescence with anti-Rex antibodies followed by laser scanner microscopy (IF-LSM) showed that Rex and Rex_a_ accumulated in the nucleus (visualized by propidium iodide, PI), while Rex_b_ and Rex_c_ were mainly localized to the cytoplasm (Figure 4a). Figure 4b shows a quantitative colocalization analysis of the Rex (X axis) and PI (Y axis) fluorescence per pixel (represented by dots in the scatter plot) that permitted calculation of the Colocalization Index (CI) indicating the fraction of Rex-positive pixels that are also PI-positive; CI values can range between 0 (no colocalization) and 1 (100% colocalization). A plot of the mean CI measured in 50 cells for each sample (Figure 4c) revealed that approximately 90% of Rex and Rex_a_ were localized in the nucleus, while less than 40% of Rex_b_ and Rex_c_ were in the nucleus.

### Functional analysis of Rex protein isoforms

**Figure 5:**
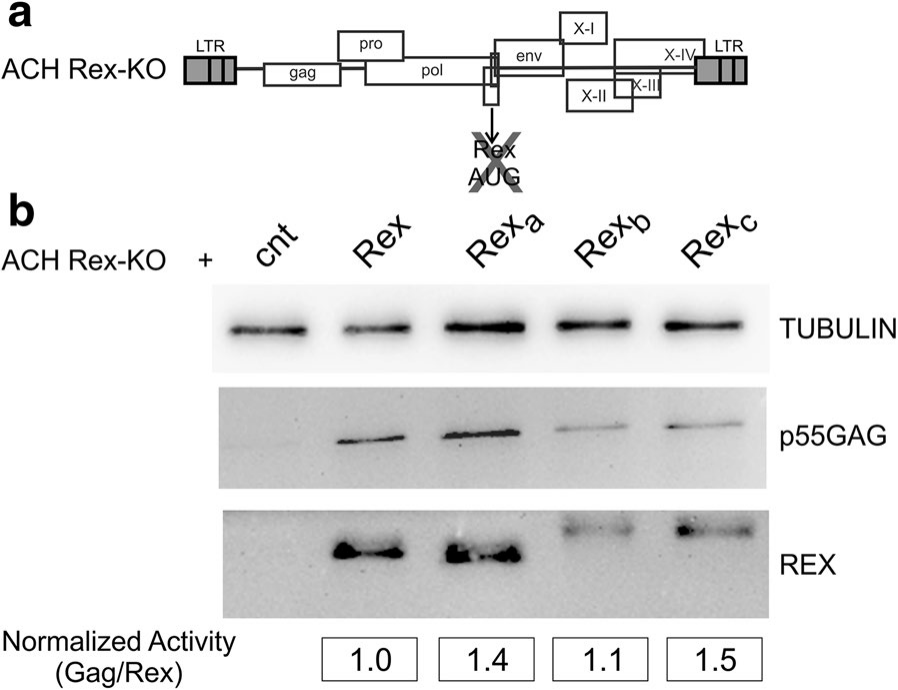
Functional analysis of Rex isoforms using a Rex-KO HTLV-1 molecular clone. **a** Schematic representation of the Rex knock-out HTLV-1 molecular clone (ACH Rex-KO) [5]. **b** Expression of the p55 Gag precursor and Rex after the co-transfection of HLtat cells with 0.5 µg of ACH Rex-KO and 0.5 µg of BlueScript (negative control, Stratagene), or pMH-Rex, pMH-Rex_a_, pMH-Rex_b_ or pMH-Rex_c_. The lower part of **b** shows the normalized Rex activity estimated by calculating the ratio between the Gag and Rex bands after subtraction of the background value obtained in the negative control sample. For immunoblot analysis, cells were harvested 24 h after transfection in Mammalian Cell Disruption Buffer (Paris-Kit, Ambion) supplemented with phosphatase inhibitors (PhosSTOP, Roche) and protease inhibitors (Complete, Roche). Protein concentration was determined by the Coomassie Protein Assay Kit (Thermo Scientific). Protein lysates (50 µg) were subjected to SDS-PAGE (12% acrylamide/bis-acrylamide) and electrotransferred to Hybond-C Extra membrane (GE Healthcare). Blots were cut into strips and blocked with 5% non-fat dry milk (Euroclone)-0.1% Tween 20-TBS (tris-buffered saline), and incubated overnight at 4°C with rabbit anti-Rex polyclonal antibody (1:5,000) [16], mouse anti-HTLV-1 p24 monoclonal antibody (1:500, Helvetica Health Care) and mouse anti-α-tubulin monoclonal antibody (1:2,000; Sigma-Aldrich) in 5% non-fat dry milk-0.1% Tween-TBS. Blots were washed and incubated for 1.5 h with horseradish peroxidase-conjugated anti-mouse or anti-rabbit secondary antibody (Pierce) diluted 1:2,500 in 5% non-fat dry milk-0.1% Tween-TBS. Blots were developed using chemiluminescence (Supersignal, Pierce) and immunoreactive bands were visualized and quantified using a Uvitec Chemiluminescence Imaging System (Cambridge) and UVIsoft Analysis software.

We next tested the ability of the Rex isoforms to rescue the expression of Gag (a Rex-dependent protein) from a Rex knock-out HTLV-1 molecular clone (ACH Rex-KO, Figure 5a) [5]. Results showed that Rex_a_, Rex_b_ and Rex_c_ were all capable of rescuing Gag expression (Figure 5b) with comparable relative activity (normalized activity, calculated as the ratio between the Gag and Rex signals).

**Figure 6:**
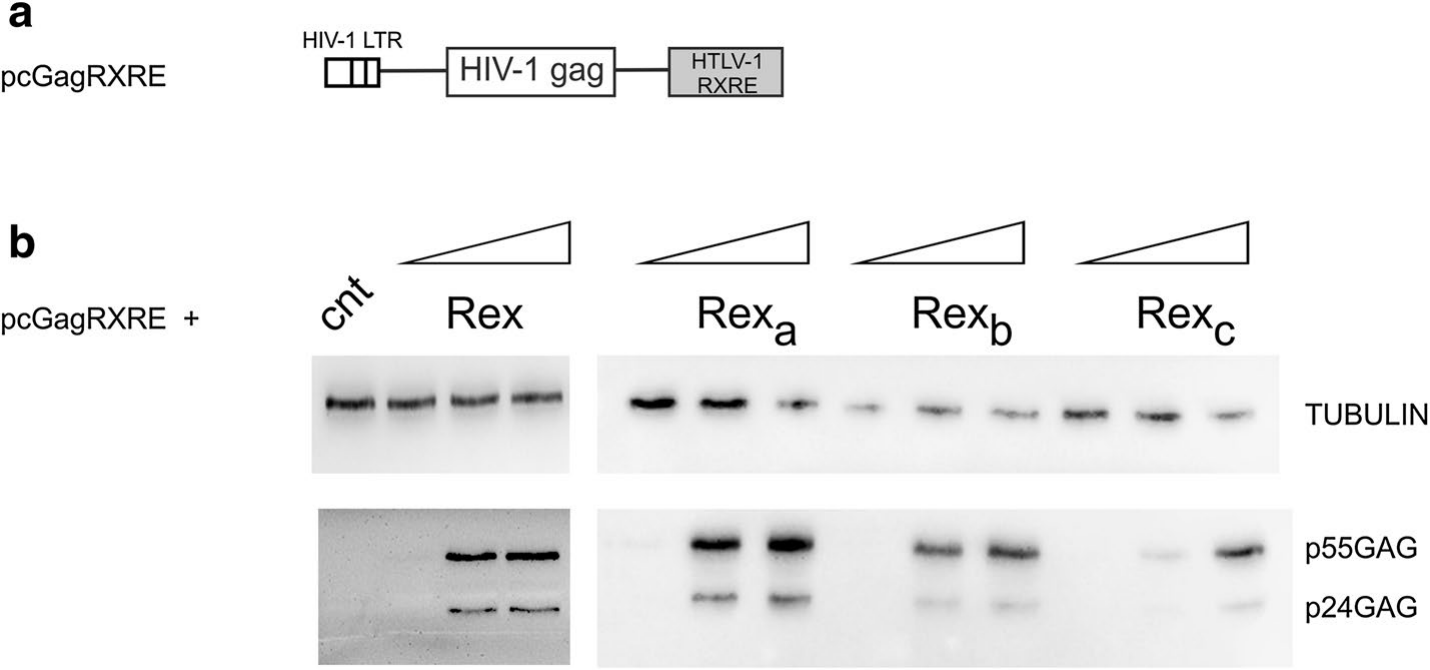
Functional analysis of Rex isoforms using the pcGagRXRE reporter plasmid. **a** Schematic representation of the pcGagRXRE reporter plasmid [17]. **b** Expression of HIV-1-p55Gag and p24Gag after the co-transfection of HLTat cells with 0.5 µg of pcGagRXRE and 0.5 µg of BlueScript (negative control) or increasing amounts (0.1, 0.2 and 0.5 µg) of pMH-Rex, pMH-Rex_a_, pMH-Rex_b_ and pMH-Rex_c_. Immunoblots were prepared and probed with mouse anti-HIV-1 p24 monoclonal antibody (1:500, Chemicon) and mouse anti-α-tubulin monoclonal antibody (1:2,000; Sigma-Aldrich) as described in the legend to Figure 5.

To validate these findings, we employed a second assay based on the pcGagRXRE reporter plasmid [11, 17], which contains the HIV-1 LTR and Gag ORF, linked to the RXRE (Figure 6a). Upon transfection of the HLtat cell line, the gag-RXRE mRNA is transcribed, but its expression is greatly impaired by cis-acting inhibitory sequences located in the *gag* gene, unless Rex is provided [18]. Cotransfection of pcGagRXRE with increasing amounts of expression plasmids coding for the Rex isoforms resulted in a dose-dependent increase in Gag expression (Figure 6b), thus confirming their functional activity.

The finding that Rex_b_ and Rex_c_ show a functional activity comparable to that of Rex and Rex_a_ suggests that their prevalent cytoplasmic localization is likely to result from increased nuclear export or decreased nuclear retention rather than an intrinsic defect in nuclear import, which would be expected to seriously impinge on their function.

The amino-acid changes in Rex_b_ and Rex_c_ do not affect the sequence of the nuclear localization signal (NLS) or the leucine-rich nuclear export signal/activation domain (NES/AD). However, the presence of 25 or 24 extra amino-acids in Rex_b_ and Rex_c_ respectively, immediately downstream of the NLS and 55 amino-acids upstream of the NES/AD (Figure 2) might induce conformational changes affecting the function of the NLS and/or the NES/AD. It is also possible that the extra residues affect the protein phosphorylation status, which is known to be important for Rex function [19], as well as subcellular localization in the case of Rex of HTLV-2 [20].

### Distinct temporal regulation of mRNAs encoding Rex isoforms

**Figure 7:**
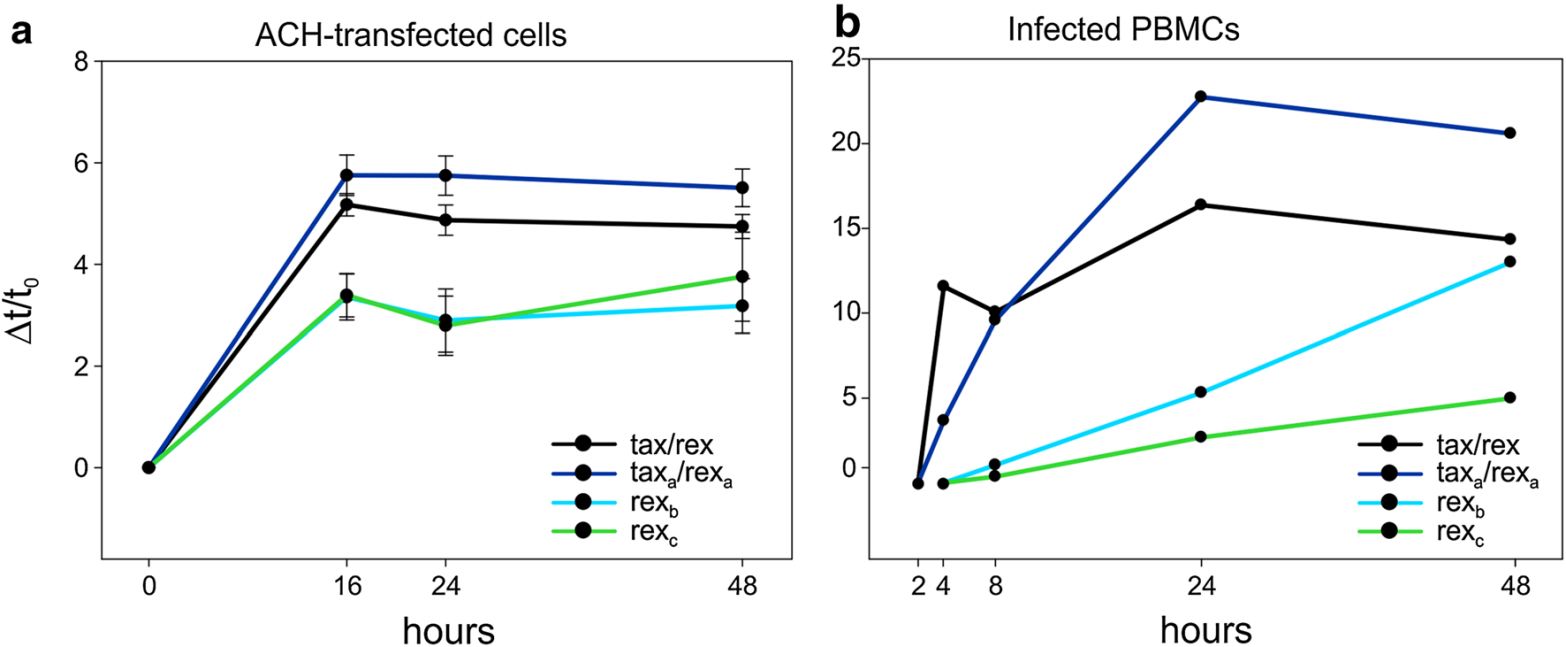
Distinct temporal regulation of transcripts encoding Rex isoforms. **a** Kinetics of expression of *tax/rex*, *tax*_*a*_ */rex*_*a*_, *rex*_*b*_ *and rex*_*c*_ mRNAs after transfection of HTLV-1 molecular clone ACH and **b** following reactivation of viral expression upon short-term culture of PBMC from the infected patient ATLL-2. X axes show the time points in hours; Y axes show the incremental variation Δt/t_0_ of the export ratio (**a**), calculated as previously described [5, 22], or of the NCN (**b**). Δt is the difference between the value measured at each time point and the value measured at the initial time point (t_0_). **a** Mean values and standard error bars from nine independent experiments.

As described above, simultaneous action of Tax and Rex at their maximal levels (as suggested by the fact that they coded by the same mRNA) is predicted to quickly shut down viral transcription due to loss of the *tax/rex* mRNA [7, 8]. It is thus necessary to assume a temporal separation between early transactivation of HTLV-1 expression by Tax and later post-transcriptional negative feedback loop mediated by Rex. In a previous study we showed that this delay could, at least in part, be attributed to an increased stability of Rex compared to Tax [5]. To test whether the delay in Rex function might also result from a distinct temporal regulation of the additional Rex-coding mRNAs, we investigated the kinetics of viral expression both following transfection of the ACH molecular clone (Figure 7a) and following reactivation of viral expression upon short-term culture of PBMC from an infected patient (Figure 7b). Both assays revealed an early selection of splice acceptors (3 and 3a) that give rise to dicistronic mRNAs encoding functional Tax and Rex isoforms. However, as the levels of the *tax/rex* and *tax*_*a*_*/rex*_*a*_ mRNAs declined, the expression of the monocistronic *rex*_*b*_ and *rex*_*c*_ mRNAs followed a steady rise, thus contributing to a prolonged duration of Rex function. These data are somewhat reminiscent of the expression strategy of HIV-1, where the two essential regulatory proteins Tat and Rev are coded by distinct mRNAs [9, 10, 21].

These findings provide insight into the mechanisms controlling HTLV-1 expression and suggest that the production of alternatively spliced monocistronic mRNAs coding for Rex isoforms contributes to the two-phase kinetics of HTLV-1 expression. The resulting pattern of viral gene expression might be important to temporally restrain the expression of highly immunogenic viral epitopes (e.g. Tax, Gag, Env), thus favouring escape from the immune response and establishment of long term persistence in the host, a key feature of HTLV-1 infection. A better understanding of how these regulatory circuits are established and maintained will shed light on the basis of viral latency and may provide groundwork for the development of new therapies for eradicating persistent infections.

## References

[1] Goncalves DU, Proietti FA, Ribas JG, Araújo MG, Pinheiro SR, Guedes AC (2010). Epidemiology, treatment, and prevention of human T-cell leukemia virus type 1-associated diseases. Clin Microbiol Rev.

[2] Currer R, Van Duyne R, Jaworski E, Guendel I, Sampey G, Das R (2012). HTLV tax: a fascinating multifunctional co-regulator of viral and cellular pathways. Front Microbiol.

[3] Nakano K, Watanabe T (2012). HTLV-1 Rex: the courier of viral messages making use of the host vehicle. Front Microbiol.

[4] Hidaka M, Inoue J, Yoshida M, Seiki M (1988). Post-transcriptional regulator (rex) of HTLV-1 initiates expression of viral structural proteins but suppresses expression of regulatory proteins. EMBO J.

[5] Rende F, Cavallari I, Corradin A, Silic-Benussi M, Toulza F, Toffolo GM (2011). Kinetics and intracellular compartmentalization of HTLV-1 gene expression: nuclear retention of HBZ mRNA. Blood.

[6] Ciminale V, Rende F, Bertazzoni U, Romanelli MG (2014). HTLV-1 and HTLV-2: highly similar viruses with distinct oncogenic properties. Front Microbiol.

[7] Cavallari I, Rende F, Bender C, Romanelli MG, D’Agostino DM, Ciminale V (2013). Fine tuning of the temporal expression of HTLV-1 and HTLV-2. Front Microbiol.

[8] Corradin A, DI Camillo B, Rende F, Ciminale V, Toffolo GM, Cobelli C (2010) Retrovirus HTLV-1 gene circuit: a potential oscillator for eukaryotes. Pac Symp Biocomput 421–432 19908394

[9] Kimata JT, Wong FH, Wang JJ, Ratner L (1994). Construction and characterization of infectious human T-cell leukemia virus type 1 molecular clones. Virology.

[10] Schwartz S, Felber BK, Benko DM, Fenyö EM, Pavlakis GN (1990). Cloning and functional analysis of multiply spliced mRNA species of human immunodeficiency virus type 1. J Virol.

[11] Ciminale V, Pavlakis GN, Derse D, Cunningham CP, Felber BK (1992). Complex splicing in the human T-cell leukemia virus (HTLV) family of retroviruses: novel mRNAs and proteins produced by HTLV type I. J Virol.

[12] Koralnik IJ, Gessain A, Klotman ME, Lo Monico A, Berneman ZN, Franchini G (1992). Protein isoforms encoded by the pX region of human T-cell leukemia/lymphotropic virus type I. Proc Natl Acad Sci USA.

[13] Rende F, Cavallari I, Romanelli MG, Diani E, Bertazzoni U, Ciminale V (2012). Comparison of the genetic organization, expression strategies and oncogenic potential of HTLV-1 and HTLV-2. Leuk Res Treat.

[14] Popovic M, Lange-Wantzin G, Sarin PS, Mann D, Gallo RC (1983). Transformation of human umbilical cord blood T cells by human T-cell leukemia/lymphoma virus. Proc Natl Acad Sci USA.

[15] Hanon E, Hall S, Taylor GP, Saito M, Davis R, Tanaka Y (2000). Abundant tax protein expression in CD4+ T cells infected with human T-cell lymphotropic virus type I (HTLV-I) is prevented by cytotoxic T lymphocytes. Blood.

[16] Bhat NK, Adachi Y, Samuel KP, Derse D (1993). HTLV-1 gene expression by defective proviruses in an infected T-cell line. Virology.

[17] Benko DM, Robinson R, Solomin L, Mellini M, Felber BK, Pavlakis GN (1990). Binding of trans-dominant mutant Rev protein of human immunodeficiency virus type 1 to the cis-acting Rev-responsive element does not affect the fate of viral mRNA. New Biol.

[18] Schwartz S, Felber BK, Pavlakis GN (1992). Distinct RNA sequences in the gag region of human immunodeficiency virus type 1 decrease RNA stability and inhibit expression in the absence of Rev protein. J Virol.

[19] Kesic M, Doueiri R, Ward M, Semmes OJ, Green PL (2009). Phosphorylation regulates human T-cell leukemia virus type 1 Rex function. Retrovirology.

[20] Narayan M, Younis I, D’Agostino DM, Green PL (2003). Functional domain structure of human T-cell leukemia virus type 2 rex. J Virol.

[21] Cavallari I, Rende F, D’Agostino DM, Ciminale V (2011). Converging strategies in expression of human complex retroviruses. Viruses.

[22] Cavallari I, Rende F, Ciminale V (2014). Quantitative analysis of human T-lymphotropic virus type 1 (HTLV-1) gene expression using nucleo-cytoplasmic fractionation and splice junction-specific real-time RT-PCR (qRT-PCR). Hum Retrovir Methods Protoc.

